# Spot Urine Estimations Are Equivalent to 24-Hour Urine Assessments of Urine Protein Excretion for Predicting Clinical Outcomes

**DOI:** 10.1155/2015/156484

**Published:** 2015-01-08

**Authors:** Boon Wee Teo, Ping Tyug Loh, Weng Kin Wong, Peh Joo Ho, Kwok Pui Choi, Qi Chun Toh, Hui Xu, Sharon Saw, Titus Lau, Sunil Sethi, Evan J. C. Lee

**Affiliations:** ^1^Department of Medicine, Yong Loo Lin School of Medicine, National University of Singapore, 1E Kent Ridge Road, Level 10 NUHS Tower Block, Singapore 119228; ^2^National University Health System, Singapore 119228; ^3^Department of Statistics and Applied Probability, Faculty of Science, National University of Singapore, Singapore 119228; ^4^Department of Laboratory Medicine, National University Health System, Singapore 119228; ^5^Department of Medicine, National University Health System, Singapore 119228; ^6^Department of Pathology, Yong Loo Lin School of Medicine, National University of Singapore, Singapore 119228

## Abstract

*Background*. The use of spot urine protein to creatinine ratios in estimating 24 hr urine protein excretion rates for diagnosing and managing chronic kidney disease (CKD) predated the standardization of creatinine assays. The comparative predictive performance of spot urine ratios and 24 hr urine collections (of albumin or protein) for the clinical outcomes of CKD progression, end-stage renal disease (ESRD), and mortality in Asians is unclear. We compared 4 methods of assessing urine protein excretion in a multiethnic population of CKD patients. *Methods*. Patients with CKD (*n* = 232) provided 24 hr urine collections followed by spot urine samples the next morning. We created multiple linear regression models to assess the factors associated with GFR decline (median follow-up: 37 months, IQR 26–41) and constructed Cox proportional-hazards models for predicting the combined outcome of ESRD and death. *Results*. The linear regression models showed that 24 hr urine protein excretion was most predictive of GFR decline but all other methods were similar. For the combined outcomes of ESRD and death, the proportional hazards models had similar predictive performance. *Conclusions*. We showed that all methods of assessments were comparable for clinical end-points, and any method can be used in clinical practice or research.

## 1. Background

The estimation of 24-hour urine protein excretion or 24-hour urine albumin excretion using urine protein to creatinine ratio (UPCR) and urine albumin to creatinine ratio (UACR), respectively, is well established in clinical practice and promulgated by practice guidelines [1]. The original studies were, however, performed prior to the standardization of creatinine assays [2–4]. The standardized creatinine values may differ by 5–20% from values obtained by methods not calibrated or traceable to the isotope dilution mass spectrometry (IDMS) standard [5]. As an example, if standardized creatinine is 20% higher than previously measured, UPCR or UACR may be 17% lower on estimation. Conversely if standardized creatinine is 10% lower, then UPCR or UACR may be 11% higher on estimation. Therefore, it is clear that creatinine standardization has important implications for the proteinuria estimation equations and their application in clinical practice. The measurement of proteinuria and albuminuria has not been standardized [6]. Moreover, the different assay methods may result in differences in the concentrations obtained. Research studies and clinical practice may favor UPCR over UACR for reason of cost, and there are no reliable methods to convert ratios [7]. However, the new iteration of the clinical practice guidelines for the identification and classification of chronic kidney disease incorporated urine albumin to creatinine ratio as part of risk stratification [8, 9]. In this study, we assessed the correlation of early morning spot urine tests to 24-hour urine protein and albumin excretion, provided conversion equations developed from a population of patients with a variety of chronic kidney disease, and compared their predictive effects for GFR decline, end-stage renal disease (ESRD), and mortality.

## 2. Methods

We used data from the Asian Kidney Disease Study as previously described [10]. Briefly, stable CKD patients were recruited from outpatient clinics for a study on glomerular filtration rates (GFR). They collected 24 hr urine collections and presented the next day for a GFR measurement and also provided early morning urine and blood samples. The 24 hr urine collection and early morning spot urine were tested for protein, albumin, and creatinine concentrations. We performed assays on the Siemens Advia 2400 (http://www.siemens.com/). Urine protein was measured using a pyrogallol-based assay calibrated to the manufacturer's internal standard. Urine albumin was measured using a PEG-enhanced immunoturbidimetric assay and was also calibrated to an internal standard. Creatinine was measured by an enzymatic method (creatininase) in a central laboratory accredited by the College of American Pathologists and the assay was calibrated with manufacturer-provided materials traceable to standardized creatinine (National Institute for Standards and Technology Standard Reference Material 967) measured by isotope dilution mass spectrometry (as recommended by the National Kidney Disease Education Program, http://www.nkdep.nih.gov/). To extract the last known serum creatinine, the hospital clinical laboratory database was reviewed. All participants were cross-referenced with our ESRD database for date of dialysis initiation or death. All longitudinal follow-up data for this study were correct till January 15, 2012. This study was approved by the National Healthcare Group, Domain-Specific Review Board (D/07/524 and 2007/00225).

## 3. Statistics

Where appropriate, variables were naturally Log-transformed before linear regression to correct for nonnormal distribution and nonconstant variability of observed points around the regression line. We used Bland-Altman analysis of agreement to assess UACR and UPCR in predicting 24 hr urine albumin excretion and 24 hr urine protein excretion, respectively. For comparisons with earlier studies, we used non-SI units in calculating the urine protein or albumin to creatinine ratios [3, 4]. Conversion equations between UACR, UPCR, 24 hr urine protein excretion, and 24 hr urine albumin excretion were developed. To assess these equations, an external dataset of 45 participants with similarly collected 24 hr urine sample followed by an early morning spot urine sample the next day was used as a validation test dataset. The predicted variable was compared to the measured values by Pearson correlation *r* and Wilcoxon signed rank test. Because clinical practice and research analysts commonly use UACR and UPCR to estimate the respective 24 hr urine excretion rates above the clinically significant thresholds of albumin >300 mg/day and protein >0.5 g/day, we also analyzed the measured (main derivation dataset, *n* = 232) and equation-predicted UACR and UPCR (validation dataset, *n* = 45) for their predictive abilities in terms of sensitivity, specificity, positive predictive value (PPV), and negative predictive value (NPV) [11, 12].

We estimated GFR using the CKD-EPI equation [13]. To compare the predictive performance of each method of urine protein or albumin assessment with GFR decline, we developed models using linear regression. We used stepwise linear regression by *P* value threshold for entry or exit from the models in a mixed direction with no rules to select the variables for models predicting GFR decline. The initial variables screened were age, gender, ethnicity, initial serum creatinine, initial serum urea, smoking history, medical history of diabetes, hypertension, coronary artery disease, peripheral artery disease, systolic blood pressure, diastolic blood pressure, body mass index, serum albumin, UACR, UPCR, 24 hr urine protein excretion, and 24 hr urine albumin excretion. The urine estimation or measurement method in the best model was substituted in turn for three additional models for comparisons. Variables with *P* values ≤0.05 and ethnicity were included in the final models. We assessed the linear regression models by reviewing the *R* ^2^, AIC (Akaike information criterion), and BIC (Bayesian information criterion) [14, 15]. Since *R* ^2^ always will increase with an increasing number of predictor variables in a model, we select our model using the AIC and BIC which penalizes models for having large number of predictor variables. AIC might favor more complex models and overfit, while BIC may select a parsimonious model and underfit. For our model assessments, the lowest AIC and BIC criteria indicate the best model. Similarly, we also assessed the predictive performance of urine protein estimations for the combined end-point of ESRD and death using Cox-proportional hazards modeling. Analyses were performed on JMP 10 (Cary, NC, USA) and R (http://www.r-project.org/).

## 4. Results

**Figure 1:**
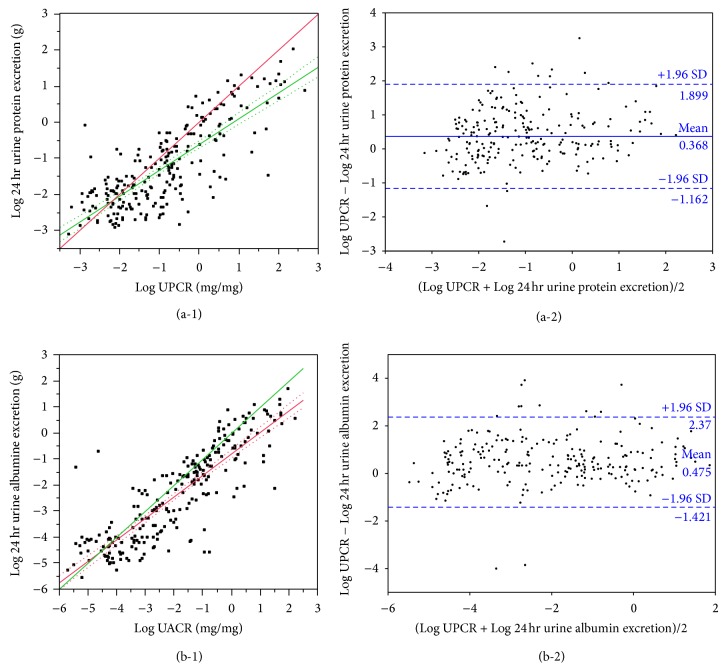
Distribution of urine estimation ratios to 24 hr urine measurements. (a-1) UPCR versus 24 hr urine protein excretion. Correlation *r* = 0.79, 95% CI 0.74–0.83. Bold line: line of identity; fine line: regression line; dotted lines: 95% CI of the regression line. (a-2) Limits of agreement of Log-transformed UPCR and 24 hr urine protein excretion. (b-1) UACR versus 24 hr urine albumin excretion. Correlation *r* = 0.86, 95% CI 0.82–0.89. Bold line: line of identity; fine line: regression line; dotted lines: 95% CI of the regression line. (b-2) Limits of agreement of Log-transformed UACR and 24 hr urine albumin excretion.

There were 232 patients with characteristics as shown in Table 1. Four patients had 24-hour urine protein excretion >3.5 g. The correlation of spot urine estimation ratios (UPCR and UACR to 24 hr urine protein and albumin excretion, resp.) is shown in Figure 1. UACR appears to predict 24 hr urine albumin excretion better as the slope is closer to 1. The prediction equations are

(1) Log  24 hr  urine  protein  excretiong  =−0.617019+0.7150918×Log  UPCRmg/mg;hhhhhhhhhhhhhhhhhhhhhhhhR2=0.64,P<0.001,Log  24 hr  urine  albumin  excretiong  =−0.800153+0.8257142×Log  UACRmg/mg;hhhhhhhhhhhhhhhhhhhhhhhh(R2=0.74,P<0.001).

**Figure 2:**
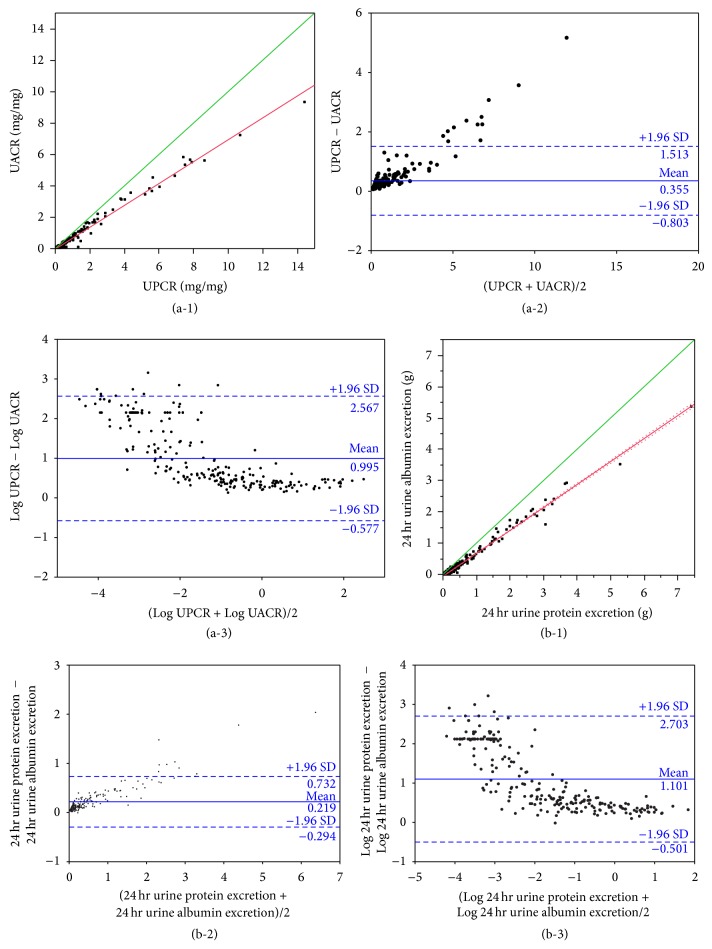
Urine albumin versus urine protein excretion. (a-1) UPCR versus UACR. Correlation *r* = 0.99, 95% CI 0.99-0.99. Bold line: line of identity; fine line: regression line; dotted lines: 95% CI of the regression line. (a-2) Limits of agreement of UPCR and UACR. (a-3) Limits of agreement of Log-transformed UPCR and UACR. (b-1) 24 hr urine protein excretion versus 24 hr urine albumin excretion. Correlation *r* = 0.99, 95% CI 0.99-0.99. Bold line: line of identity; fine line: regression line; dotted lines: 95% CI of the regression line. (b-2) Limits of agreement of 24 hr urine protein excretion and 24 hr urine albumin excretion. (b-3) Limits of agreement of Log-transformed 24 hr urine protein excretion and 24 hr urine albumin excretion.

UPCR has poorer correlation to 24 hr urine protein excretion (*r* = 0.80, 95% CI 0.75–0.84) than UACR has to 24 hr urine albumin excretion (*r* = 0.86, 95% CI 0.82–0.89). UACR is highly correlated to UPCR (*r* = 0.99, 95% CI 0.99-0.99) (Figure 2). 24 hr urine albumin excretion is also highly correlated to 24 hr urine protein excretion (*r* = 0.99, 95% CI 0.99-0.99). UACR is less correlated with 24 hr urine protein excretion (*r* = 0.78, 95% CI 0.72–0.83) than UPCR is with 24 hr urine albumin excretion (*r* = 0.81, 95% CI 0.76–0.85). The sensitivity and specificity of UACR of between 30 and 300 mg/mg for predicting 24 hr urine albumin excretion of between 30 and 300 mg (microalbuminuria) are 0.69 and 0.77, respectively. The PPV and NPV are 0.63 and 0.81, respectively. The sensitivity and specificity of UACR of >300 mg/mg for predicting 24 hr urine albumin excretion of >300 mg (macroalbuminuria) are 0.94 and 0.84, respectively. The PPV and NPV are 0.70 and 0.97, respectively. The sensitivity and specificity of UPCR of >0.5 mg/mg for predicting 24 hr urine protein excretion of >0.5 g/day are 0.90 and 0.81, respectively. The PPV and NPV are 0.66 and 0.95, respectively.

The various conversion equations derived from the main study data (*n* = 232) are in Table 2. Using the external validation dataset (*n* = 45) for assessing the performance of the conversion equations, predicted spot urine ratios were highly correlated but significantly differed from measured spot urine values. UPCR correlated with “predicted 24 hr urine protein excretion” (*r* = 0.63, 95% CI 0.41–0.78) better than UACR with “predicted 24 hr urine albumin excretion” (*r* = 0.55, 95% CI 0.31–0.73). The sensitivity and specificity of “predicted-UACR,” calculated from UPCR, of >300 mg/mg for predicting 24 hr urine albumin excretion of >300 mg (macroalbuminuria) are 0.059 and 1.0, respectively. The PPV and NPV are 1.0 and 0.64, respectively. The sensitivity and specificity of “predicted-UACR,” calculated from 24 hr urine protein excretion, of >300 mg/mg for predicting 24 hr urine albumin excretion of >300 mg (macroalbuminuria) are 1.0 and 0.89, respectively. The PPV and NPV are 0.85 and 1.0, respectively. The sensitivity and specificity of “predicted-UPCR,” calculated from UACR, of >0.5 mg/mg for predicting 24 hr urine protein excretion of >0.5 g/day are 0.1 and 1.0, respectively. The PPV and NPV are 1.0 and 0.58, respectively.

There were 225 patients with available follow-up serum creatinine to determine estimated GFR decline. By stepwise linear regression, we developed 4 final models to compare the performance of UACR, UPCR, and 24 hr urine protein or albumin excretion (Table 3). In all models, the method of urine protein or albumin assessment and the initial serum urea were significant. The model containing 24 hr urine protein excretion predicted GFR decline best. All the models had good predictive performance for GFR decline, with the best performance in the model that included 24 hr urine protein excretion, followed in order by 24 hr urine albumin excretion, UPCR, and UACR.

The median follow-up was 37 months (IQR 26–41). There were 19 patients who reached ESRD (9/19, 47% women) and 9 (3/9, 33% women) who died during the follow-up period (4 had ESRD before dying). Patients who reached ESRD were of similar age (59.8 ± 10.3 versus 58.8 ± 13.0 years) but had a higher serum creatinine (311 ± 113 versus 138 ± 75 *μ*mol/L) and 24 hr urine albumin (1515 ± 1251 versus 283 ± 504 mg). We created 4 Cox proportional hazard models to compare the performance of the various proteinuria assessments for predicting the combined end-point of ESRD and death (Table 4). All models, which included the standard adjusters (age, gender, and ethnicity), were significant for all methods of proteinuria assessment (all *P* < 0.001) and Log- serum urea (all *P* < 0.001).

## 5. Discussion

This is the first prospective study of a multiethnic Asian population with a wide variety of CKD (diabetic and nondiabetic) patients that simultaneously evaluates early morning spot urine prediction ratios to 24 hr urine collections, while accounting for the standardization of the creatinine assay. Previous studies were retrospective and did not have simultaneous collection of spot urine and 24 hr urine collections [16]. Clinical practice and research involving CKD patients are highly dependent on the use of urine protein or albumin to creatinine ratios as estimates of their respective 24 hr urine excretions. Yet, many are unaware of the implications of the use of urine ratios [17, 18]. Fundamentally, the main questions that need to be answered are as follows. (1) Does UACR or UPCR predict 24 hr urine albumin or protein excretion? (2) Which spot urine ratio predicts 24 hr urine excretion better? (3) In our setting, how do the ratios relate to the 24 hr urine excretions? Especially, now that we have standardized creatinine assays but not for albumin and protein assays. And, of course, (4) which parameter (ratios or 24 hr urine excretions) predicts longitudinal outcomes data better (GFR declines, ESRD, or mortality in CKD patients)?

Our study shows that UACR is correlated to 24 hr urine albumin excretion better than UPCR. We did not find as high a correlation for UPCR as the earlier studies [3, 4]. This may be partly due to creatinine calibration which results in a systematically larger ratio. The clinical practice guideline for the identification and classification of CKD incorporates UACR in addition to estimated GFR for staging CKD. Our study derived the conversion equations for clinical research or practice needing spot or 24 hr urine albumin or protein excretions, specific to the assay methods. On average, predicted spot urine ratios were reasonably correlated but were significantly different from measured values. Many clinical and research databases contain both UACR and UPCR in the same patients; analysts often apply conversion equations for the purposes of analysis. But the sensitivity of “predicted UACR” or “predicted UPCR” for identifying clinically significant 24 hr urine excretion rates is poor. Therefore, we do not recommend using the conversion equations of UACR to UPCR, and vice versa. However, the performance of “predicted-UACR” calculated from 24 hr urine protein excretion appears to be acceptable for identifying clinically significant proteinuria of >0.5 g/day. Nonetheless, in clinical practice, it is currently not recommended to interchangeably convert urine albumin and urine protein concentrations [11].

Contrary to the findings by Ruggenenti et al. in their cohort of only nondiabetic patients, we did not find that UPCR predicted GFR decline better than 24 hr urine protein excretion [4]. But, in all our models, all methods of assessing urine protein excretion rates were significant for predicting GFR decline, ESRD, and mortality. In our opinion, this supports the current practice of using spot urine tests as estimates of assessing 24 hr urine protein or albumin excretion [11, 16]. And all of the methods of assessments are significantly associated with predicting clinical outcomes in a multiethnic Asian population with different types of CKD, making our results more generalizable and supportive of clinical practice and research.

The strengths of our study include a fairly large multiethnic Asian population comprising of both diabetic and nondiabetic CKD, with systematically collected spot and 24 hr urine when compared to previous studies [3, 4]. We also used turbidimetry, a robust method for determining albumin concentrations, although some others advocate using nephelometry as the preferred albumin assay method [7]. Our study is also limited by fewer CKD patients with nephrotic-range proteinuria (24 hr urine protein excretion >3.5 g). However, it had been shown that spot urine estimates were less accurate at higher levels of proteinuria [3, 4]. The urine estimation to 24 hr measurements may be less accurate since the urine collections are self-directed. Conversely, others would also argue that the derived equations are more reflective of actual practice, and, therefore, prediction equations and longitudinal analyses will be more valid and generalizable to clinical practice. Moreover, in practice, we are generally interested in categories of proteinuria excretion, namely, <1 g/day, 1 to 3 g/day, and >3 g/day, and that, at higher levels, one should obtain a 24 hr urine collection to ascertain the parameters for initiating treatment of CKD. The sample size limits the accuracy of the multivariate regression models, and further definitive studies are required.

## 6. Conclusions

In summary, we appraised the use of urine spot ratios for assessing urine protein excretion rates and developed helpful conversion equations for both clinical research and practice. We showed that all methods of urine protein assessment were comparable for clinical end-points, and any method can be used in clinical practice or research.

## Figures and Tables

**Table 1:** Characteristics of participants.

Age (years)	58.4 ± 12.8
Male (*n*, %)	120 (51.7)
Ethnicity (*n*, %)	
All	232 (100)
Chinese	94 (40.5)
Malay	74 (31.9)
Indian	56 (24.1)
Others	8 (0.03)
Height (m)	1.59 ± 0.09
Weight (kg)	70.3 ± 15.9
Body mass index (kg/m^2^)	27.6 ± 5.5
Body surface area (m^2^)	1.72 ± 0.21
Measured GFR (mL/min/1.73 m^2^)	51.7 ± 27.5
Serum creatinine (µmol/L)	153 ± 92
Serum protein (g/L)	72.2 ± 5.7
Serum albumin (g/L)	41.8 ± 3.2
Serum urea (mmol/L)	8.35 ± 6.35
24 hr urine volume (L)	1.76 ± 0.78
24 hr urine protein (g)	0.6 ± 0.9
24 hr urine albumin (mg)	383.7 ± 685.9
24 hr urine creatinine (mmol)	8.2 ± 3.6
Spot urine protein (g/L)	0.64 ± 1.04
Spot urine albumin (mg/L)	413.3 ± 721.5
Spot urine creatinine (mmol/L)	6.9 ± 4.6
UPCR (mg/mg)	1.03 ± 1.87
UACR (mg/mg)	0.68 ± 1.3
Diabetes (*n*, %)	119 (51)
Hypertension (*n*, %)	192 (83)
Cause of CKD (*n*, %)	
Hypertension	115 (49.6)
Diabetic nephropathy	54 (23.3)
Glomerular disease	38 (16.4)
Polycystic kidney disease	6 (2.6)
Obstructive kidney disease	4 (1.7)
Other or unknown cause	15 (6.5)

Data shown as mean ± SD or frequency (percentage).

**Table:** (a) Conversion predicting equations

Equations	*P* value
Log ⁡24 hr urine protein excretion (g) = −0.617019 + 0.7150918 × Log UPCR (mg/mg)	<0.001
Log ⁡24 hr urine albumin excretion (g) = −0.800153 + 0.8257142 × Log UACR (mg/mg)	<0.001
Log UACR (mg/mg) = −0.656352 + 1.3881178 × Log UPCR (mg/mg)	<0.001
Log UPCR (mg/mg) = 0.3216439 + 0.6394674 × Log UACR (mg/mg)	<0.001
Log UACR (mg/mg) = −0.270587 + 1.2870223 × Log ⁡24 hr urine protein excretion (g)	<0.001

**Table:** (b) Performance of conversion equations using an external validation dataset (*n* = 45)

Predictor	Predicted variable	Predicted value	Measured value	*P* value	Correlation (95% CI)
UPCR (mg/mg)	24 hr urine protein excretion (g)	0.74 (0.67–0.82)	0.38 (0.14–0.92)	0.0051	0.63 (0.41–0.78)
UACR (mg/mg)	24 hr urine albumin excretion (g)	0.91 (0.87–0.94)	0.21 (0.02–0.58)	<0.001	0.55 (0.31–0.73)
UPCR (mg/mg)	UACR (mg/mg)	0.01 (0.00–0.03)	0.03 (0.00–0.08)	<0.001	0.97 (0.95–0.98)
UACR (mg/mg)	UPCR (mg/mg)	0.15 (0.04–0.29)	0.05 (0.02–0.13)	<0.001	0.95 (0.91–0.97)
24 hr urine protein excretion (g)	UACR (mg/mg)	0.22 (0.06–0.69)	0.03 (0.00–0.08)	<0.001	0.81 (0.68–0.89)

Reported as median (25th–75th percentile); *P* value of the difference between predicted and measured values; Pearson correlation *r* (95% confidence interval).

**Table 3:** Models predicting GFR decline^*^.

	*R* ^2^	95% CI	Estimate	Variables	*P* value	AIC	BIC
Model 1	0.10	0.028 to 0.172	−0.211	Log 24 hr urine protein excretion	<0.001	524.97	545.65
			0.311	Log serum urea	0.001		

Model 2	0.095	0.025 to 0.166	−0.127	Log 24 hr urine albumin excretion	<0.001	526.13	546.81
			0.291	Log serum urea	0.002		

Model 3	0.071	0.009 to 0.133	−0.148	Log UPCR	0.001	532.38	553.06
			0.289	Log serum urea	0.003		

Model 4	0.066	0.005 to 0.126	−0.092	Log UACR	0.002	533.65	554.33
			0.275	Log serum urea	0.0072		

^*^All models adjusted for ethnicity.

**Table 4:** Proportional hazards models predicting the combined end-point of ESRD and death.

	Model	*P* value	Term	*P* value	Risk ratio	Lower 95% CI	Upper 95% CI
	−Log likelihood						
Model 1	−79.45	<0.001	Log 24 hr urine protein	<0.001	3.87	2.37	6.31
			Log serum urea	<0.001	17.62	6.27	49.54
			Age	0.225	0.97	0.93	1.02
			Gender	0.310	1.66	0.62	4.41
			Malay ethnicity	0.155	2.08	0.76	5.72
			Indian and others ethnicity	0.572	1.43	0.41	4.93

Model 2	−80.03	<0.001	Log 24 hr urine albumin	<0.001	2.90	1.87	4.51
			Log serum urea	<0.001	16.94	6.25	45.89
			Age	0.28	0.98	0.94	1.02
			Gender	0.31	1.65	0.62	4.40
			Malay ethnicity	0.16	2.07	0.75	5.69
			Indian and others ethnicity	0.63	1.36	0.39	4.72

Model 3	−77.30	<0.001	Log UPCR	<0.001	3.82	2.40	6.07
			Log serum urea	<0.001	23.52	7.60	72.85
			Age	0.07	0.96	0.92	1.00
			Gender	0.01	3.89	1.33	11.40
			Malay ethnicity	0.19	1.94	0.72	5.22
			Indian and others ethnicity	0.40	1.71	0.48	6.04

Model 4	−79.38	<0.001	Log UACR	<0.001	2.98	1.93	4.58
			Log serum urea	<0.001	20.67	7.09	60.23
			Age	0.12	0.97	0.92	1.01
			Gender	0.03	3.23	1.15	9.12
			Malay ethnicity	0.18	1.96	0.73	5.24
			Indian and others ethnicity	0.42	1.69	0.48	6.05

All models included the standard terms of age, gender, and ethnicity. Risk ratios for continuous variables are per unit change in the regressor, and for categorical variables: gender (men/women), with women being the reference level.
